# A comparison between barbed suture and conventional suture in total knee arthroplasty: a systematic review and meta-analysis

**DOI:** 10.1186/s42836-020-00028-6

**Published:** 2020-03-27

**Authors:** Erliang Li, Wenjing Niu, Tingting Lu, Xiaolin Li, Tong Zhang, Jinchi Cai, Wenji Wang

**Affiliations:** 1 The First Clinical Medical College of Lanzhou University, Lanzhou, 730000 China; 2 Department of Orthopedics, The First Hospital of Lanzhou University, Lanzhou, 730000 China; 3 The Second Affiliated Hospital of Air Force Military Medical University, Xi’an, 710000 China; 4 GRADE Working Group China Center, School of Basic Medical Sciences, Lanzhou University, Lanzhou, 730000 China

**Keywords:** Barbed suture, Wound suture, Total knee arthroplasty, Systematic reviews, Meta-analyses

## Abstract

**Objective:**

The aim of this systematic review and meta-analysis was to evaluate the efficacy of barbed versus conventional sutures in total knee arthroplasty.

**Methods:**

Two investigators independently performed data extraction and assessed study quality using the keywords “barbed suture, wound suture, total knee arthroplasty” in two search trials, individual trials, and trials from Systematic Reviews or Meta-analyses in PubMed, Cochrane Library, Web of Science, and EMBASE databases.

**Result:**

A total of 11 articles (involving 1546 total knee arthroplasties) were included in this study. Comparison was made between barbed and conventional sutures in terms of various measures. No significant differences were identified in superficial infection and deep infection (*p* > 0.51; odds ratio 0.84 [95% confidence interval, 0.50, 1.4] and *p* > 0.28; odds ratio 0.50 [95% confidence interval, 0.14, 1.75], respectively). There was no significant difference in time for capsular suture (*p* < 0.05; odds ratio − 4.05 [95% confidence interval, − 4.39, − 3.71]). There existed no significant differences in Hospital for Special Surgery Knee Score and Knee Society Score (*p* > 0.05; odds ratio − 1.20 [95% confidence interval, − 2.98, 0.58] and *p* > 0.05; odds ratio − 1.62 [95% confidence interval, − 4.06, 0.18], respectively). No significant differences were revealed in suture breakage and needle stick injury (*p* < 0.05; odds ratio 36.51 [95% confidence interval, 7.06, 188.72] and *p* < 0.05; odds ratio 0.16 [95% confidence interval, 0.04, 0.72], respectively). No significant difference was exhibited in dehiscence (*p* = 0.99; odds ratio 0.99 [95% confidence interval, 0.41, 2.38]).

**Conclusion:**

In total knee arthroplasty, both barbed and conventional sutures yielded similar results in terms of superficial and deep infection, Hospital for Special Surgery Knee Score, Knee Society Score, and wound dehiscence. Barbed suture was associated with higher incidence of suture breakage, shorter suture time, and less needle stick injury.

## Introduction

A total knee arthroplasty (TKA) involves replacement of all three compartments of the diseased knee joint [1]. According to the American Academy of Orthopaedic Surgeons (AAOS 2019), more than 600,000 TKAs are performed annually in the United States [2]. Surgical site wound closure plays a vital role in postoperative success, but researchers haven’t reached a consensus about the the optimal strategy [3].

Wound closure involves the use of sutures in an interrupted, layered closure, with or without the use of skin staples [4]. Traditionally, simple interrupted suture is regarded as most appropriate for wounds with well-approximated skin edges under no tension [5]. Ideally, a suture material should have minimal tissue reactivity but provide a longer period of effective wound support [6]. A meta-analysis of 10 controlled trials showed that the absorbable sutures worked comparably as non-absorbable sutures for wound closure in the cases of wound infection and other complications [7]. In a retrospective study of 181 patients, Newman *et al* [8] found that use of staple was associated with fewer complications than use of suture. However, more studies concluded that nylon sutures and skin staples had similar wound complication rates, patient satisfaction with wound appearance, and cosmesis scores [9]. Up to now, optimal suture method for TKA remains a matter of debate with contradictory results.

This systematic review and meta-analysis evaluated efficacy of barbed *versus* conventional sutures in TKA in terms of wound infection, suturing time, and postoperative knee joint function. The hypothesis of the study was that staples are not significantly different from sutures in clinical outcomes for skin closure in TKA.

## Materials and methods

### Search strategy

We electronically searched keywords “barbed suture, wound suture, and total knee arthroplasty” in two search trials, individual trials, in PubMed, Cochrane Library, Web of Science, and EMBASE databases from June 20, 2019 to July 22, 2019. We also searched “systematic reviews and meta-analyses” in PubMed, Cochrane Library, Web of Science, and EMBASE databases to retrieve systematic reviews or meta-analyses.

### Inclusion and exclusion criteria

Our eligibility criteria included: 1) English language articles; 2) full-text articles; 3) systematic reviews or meta-analyses on skin closure in TKA; 4) a randomized controlled comparison study; and 5) barbed suture *versus* traditional suture. Our exclusion criteria were: 1) cadaver or animal studies; 2) comments or letter; and 3) partial articles.

### Risk-of-bias assessments

The methodological quality of the included randomized controlled trials (RCTs) was independently assessed by two investigators (EL and NJ) according to the Cochrane Risk of Bias Criteria (version 6, update September 2018). The Cochrane Handbook for Systematic Reviews of Interventions (http://training.cochrane.org/handbook) provided guidance for preparation of Cochrane Intervention reviews. Each quality item was graded as low risk, high risk, or unclear risk. The seven items used to evaluate bias in each trial included generation of a random sequence, allocation concealment, blinding of participants and staff, blinding of outcome assessment, incomplete outcome data, selective reporting, and other biases. Other biases were defined as trials that could bias the results by sponsorship and trials that did not have similar baseline characteristics between the different intervention groups. The non-RCT experiments were assessed by two investigators (EL and LT) according to the Newcastle-Ottawa Scale. A total of 8 items were rated with a 9-star rating system, and a rating greater than 7 stars was used as criterion for inclusion in this study (http://www.ohri.ca/programs/clinical_epidemiology/oxford.asp).

### Data extraction

Two investigators (LL and ZT) independently extracted the systematic reviews or meta-analyses of RCT studies. Upon merging with the studies retrieved by individual trials, two investigators (LL and LT) independently extracted the combined studies. The extracted information included lead author, publication year, study type, country, number of total cases, gender, age, body mass index, surgical application and clinical outcomes.

### Statistical analysis

For dichotomous variables, we used Mantel-Haenszel method and odds ratio with fixed effect model, to summarize the outcomes of barbed suture and traditional suture for TKA. Statistical heterogeneity between the pooled data was evaluated using I^2^ statistic, and when I^2^ > 40% or when subgroup analysis was required, random effect model was used. For continuous variables, we employed inverse-variance and fixed-effect meta-analyses. I^2^ statistic was used to evaluate statistical heterogeneity between pooled data. When I^2^ > 40% or when a subgroup analysis was required, random effect model was used. When more than 10 studies were included, funnel plots were used to identify publication bias and other biases. When the Knee Society Score assessment time was inconsistent, subgroup analysis was performed, and the random effect model was used. All meta-analyses were conducted using RevMan version 5.3. A *p* < 0.5 was considered statistically significant.

## Results

**Fig. 1:**
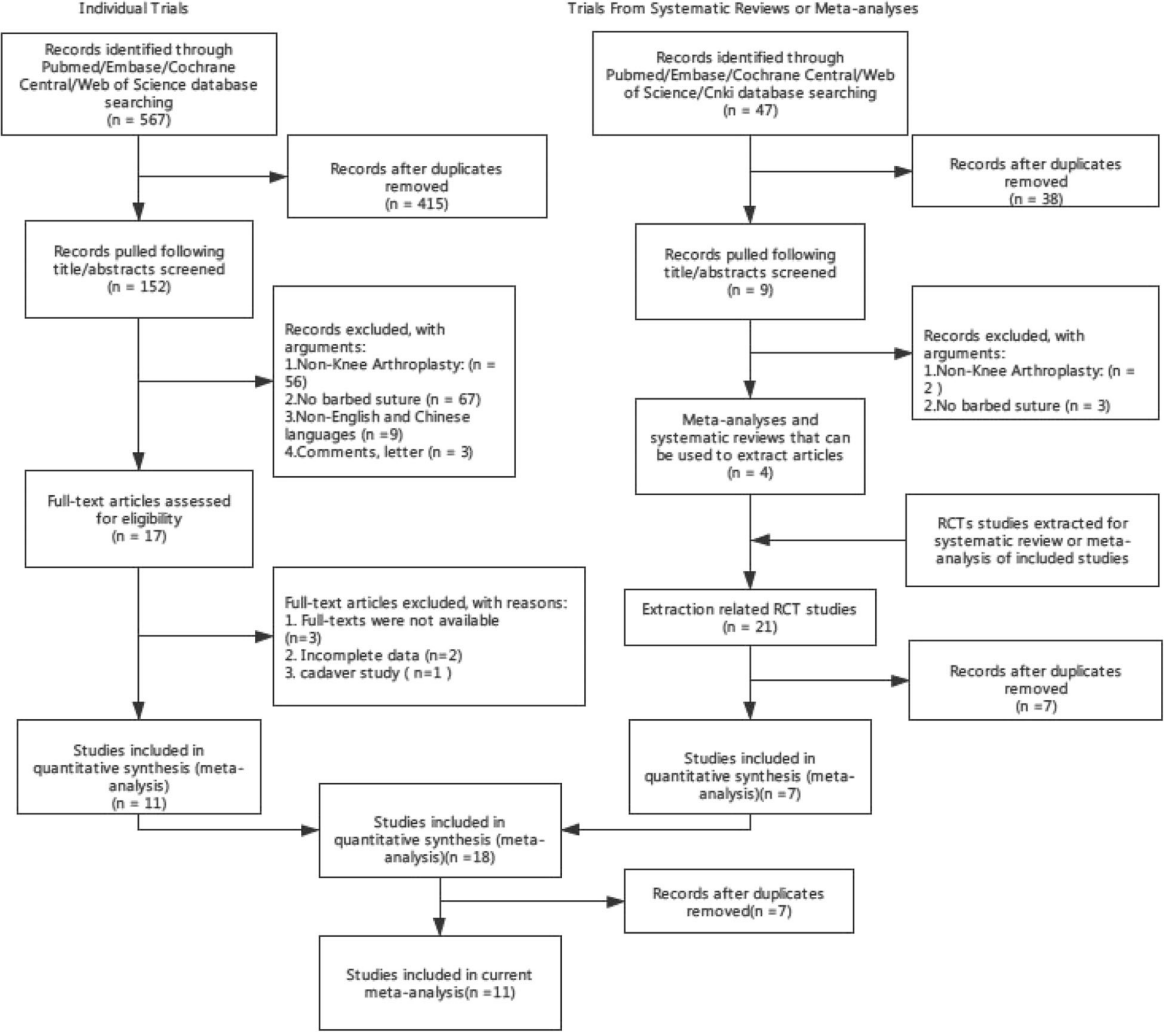
Flow chart of search strategy

**Fig. 2:**
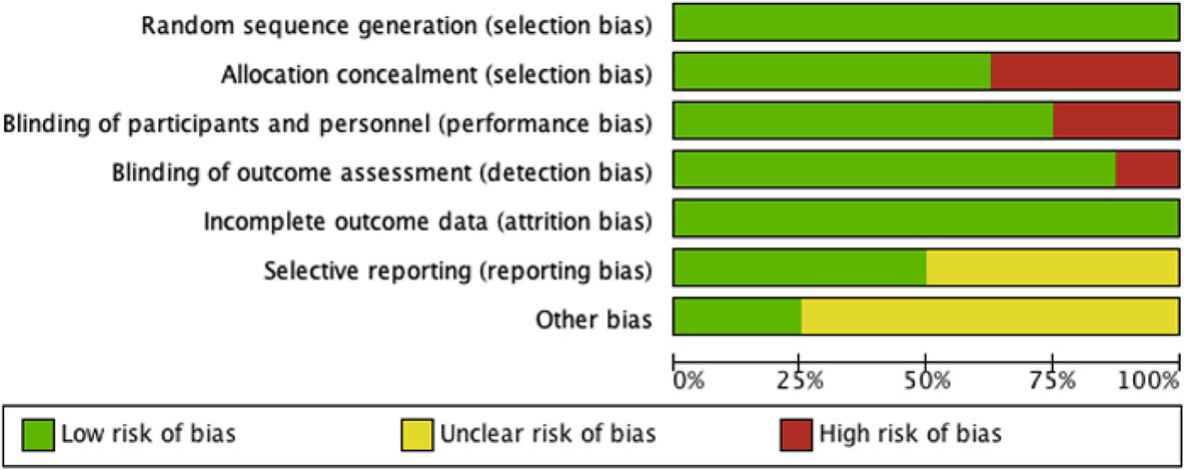
Risk of bias assessment graph for randomized controlled trials

**Fig. 3:**
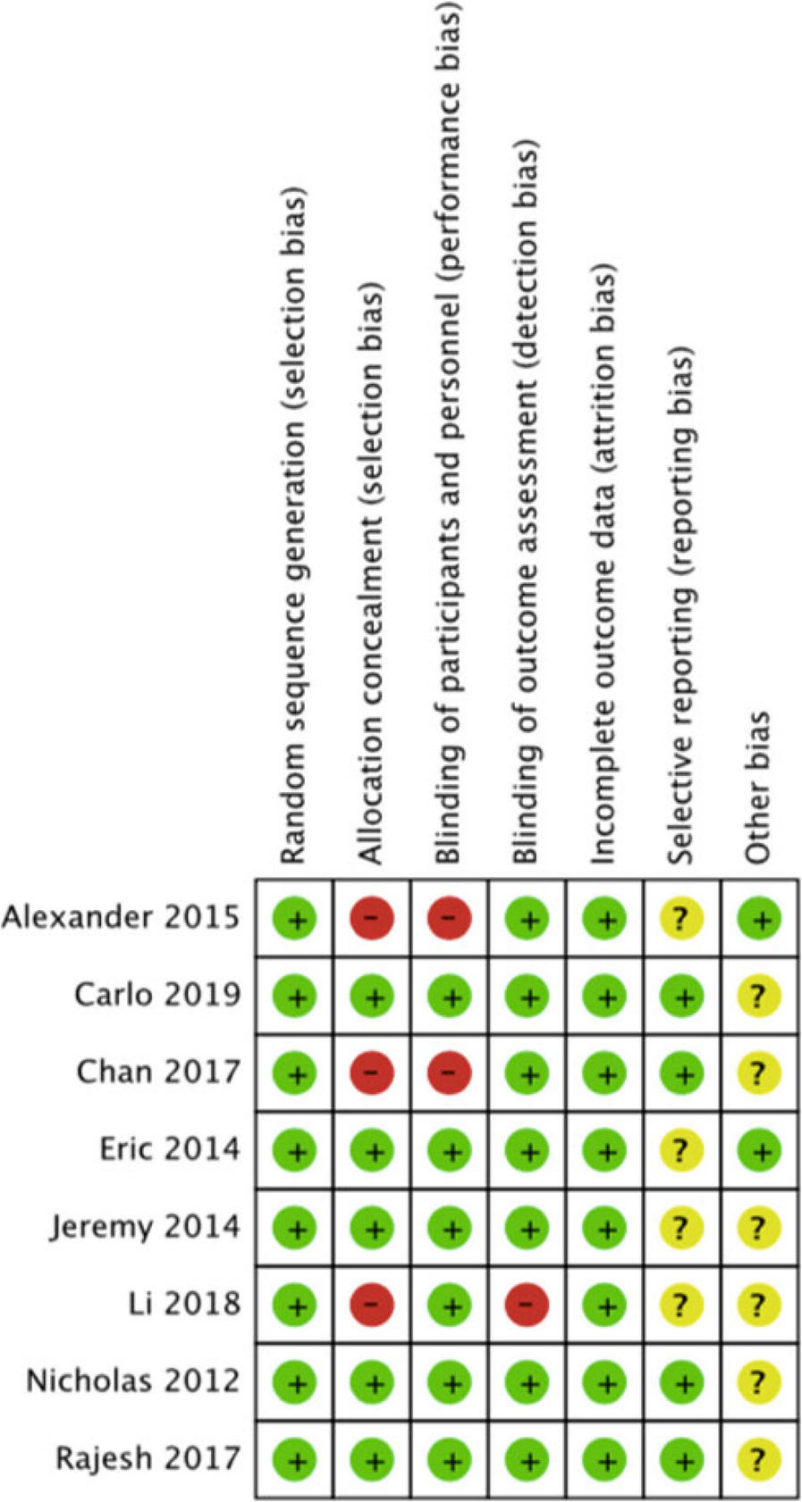
Risk of bias summary for randomized controlled trials

**Fig. 4:**
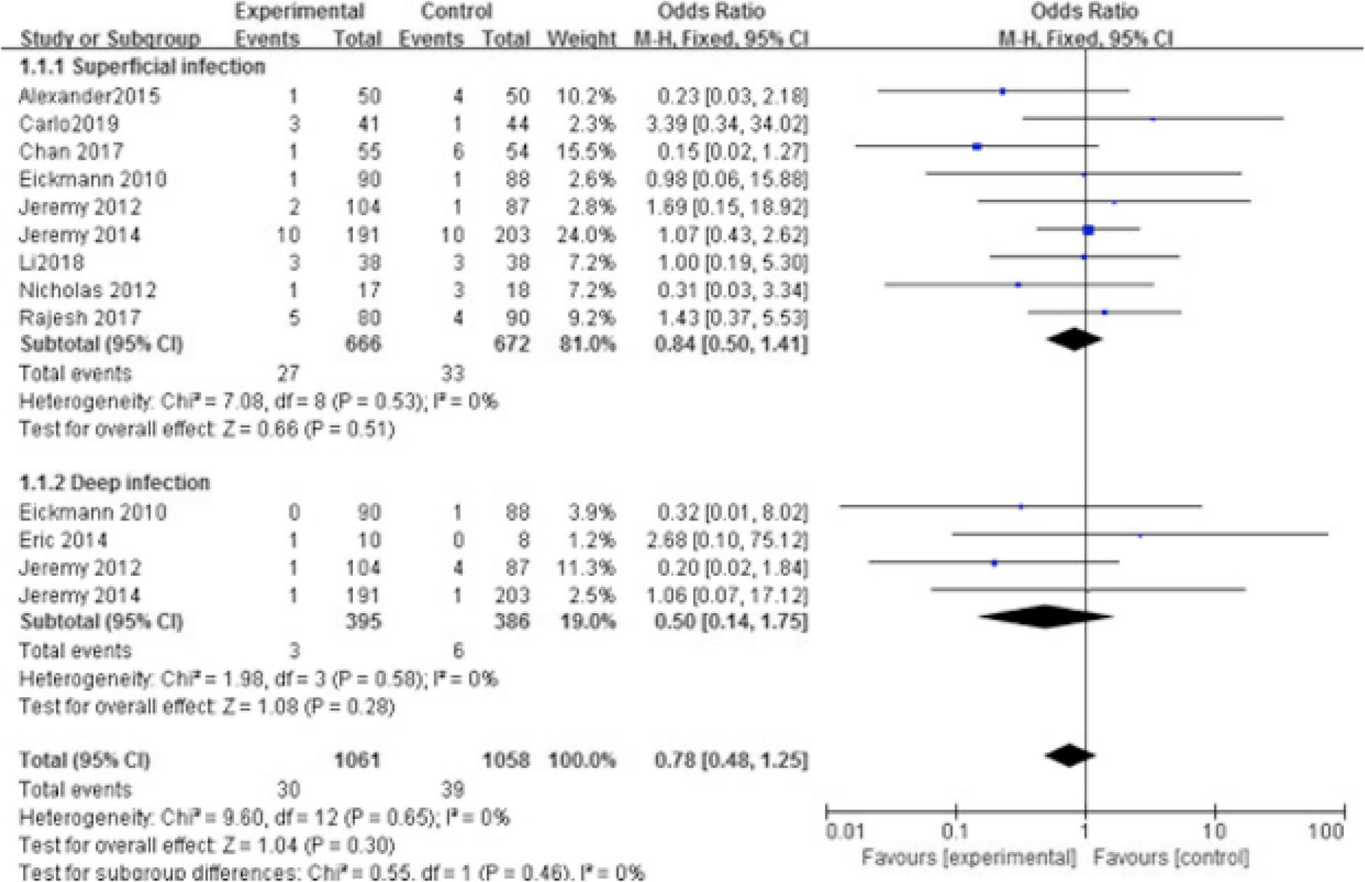
Meta-analysis of superficial and deep infection rates of barbed *versus *conventional sutures. There was no significant difference (OR 0.84 [95% CI, 0.50, 1.41]) between barbed sutures (666 cases) and conventional sutures (672 cases) with regards to the incidence of superficial infection. There was no significant difference (OR 0.50 [95% CI, 0.14, 1.75]) between barbed sutures (395 cases) and conventional sutures (386 cases) with regards to the incidence of deep infection. Overall infection was not significantly associated with barbed *versus* conventional sutures (OR 0.78 [95% CI, 0.48, 1.25]) (Overall effect, *p* = 0.03)

**Fig. 5:**
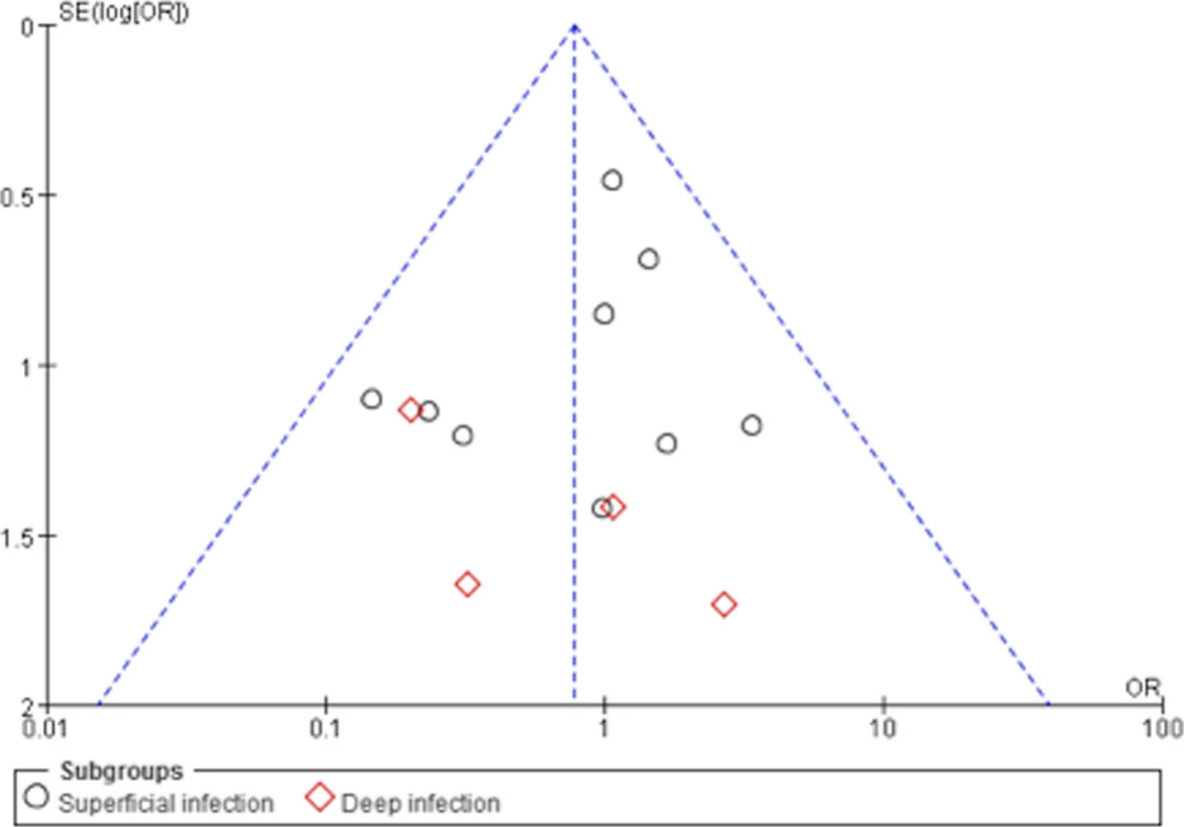
The results of funnel plot are based on the incidence of infection, which shows distribution of each study in the funnel. Symmetrical distribution suggests no publication bias

**Fig. 6:**
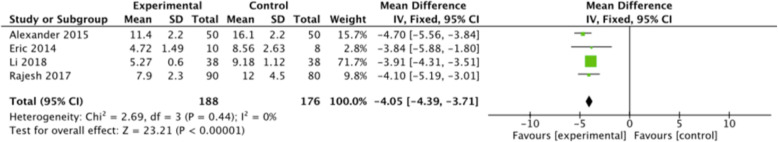
Meta-analysis of time for capsular suture using barbed *versus* conventional sutures. There was a significant correlation (OR -4.05 [95% CI, − 4.39, − 3.71]) (Test for overall effect, *p* < 0.00001) between barbed suture (188 cases) and traditional suture (176 cases)

**Fig. 7:**
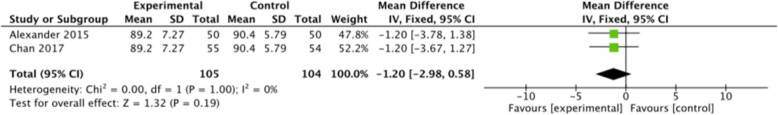
Meta-analysis of barbed *versus* conventional suture based on Hospital for Special Surgery knee questionnaire. In two studies, barbed suture (105 cases) *versus* conventional suture (104 cases) showed no significant association (OR -1.20 [95% CI, − 2.98, 0.58]) (Test for overall effect, *p* = 0.19) with regards to Hospital for Special Surgery score

**Fig. 8:**
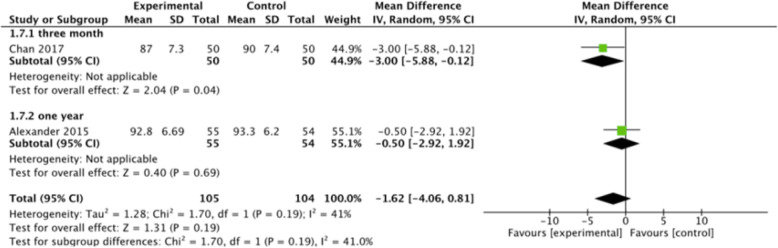
Meta-analysis of barbed *versus* conventional suture based on Functional Scoring System. There was no significant correlation (OR -1.62 [95% CI, − 4.06, 0.18]) (Test for overall effect, *p* = 0.19)

A total of 614 studies (covered by 47 systematic reviews or meta-analyses) were retrieved by two search approaches. After pooling, 11 studies were finally included according to the inclusion and exclusion criteria (Fig. 1). There were 8 RCTs and 3 non-RCTs, with a total of 1546 TKAs from USA [4, 10–15], China [16, 17], India [18], and Spain [19]. Surgical approach, time for capsule suture, superficial wound infection, deep wound infection, wound dehiscence, suture breakage, needlestick, Hospital for Special Surgery (HSS) [20] knee questionnaire, and Functional Scoring System (KSS) [21] are shown in Table 1. Risks of bias assessment of eight RCT studies are shown in Figs. 2 and 3. Risks of bias assessment of three non-RCT studies are shown in Table 2. Meta-analyses of barbed *versus* conventional sutures are shown in Figs. 4, 5, 6, 7 and 8.

**Table 1:** Demographic Characteristics and Surgical Approach

Included Trials	type study	country	total	Gender (F/M)		Age (years)		BMI (kg/m2)		Surgical Approach	Clinical Outcomes
				T	C	T	C	T	C		
Eickmann, 2010 [13]	retrospective study	USA	178	54/32	56/23	67.6 ± 10	68 ± 9.7	N	N	N	2, 3, 4
Jeremy, 2012 [4]	retrospective study	USA	191	66/32	55/30	61 ± 11	63 ± 11	32 ± 7	33 ± 7	medial parapatellar	3, 4
Nicholas, 2012 [12]	RCT	USA	35	N*	N*	N*	N*	N*	N*	N	2
Jeremy, 2014 [11]	RCT	USA	394	114/77	126/77	64 ± 10	63 ± 10	33 ± 8	33 ± 8	medial parapatellar	2, 3, 5, 6
Eric, 2014 [10]	RCT	USA	18	5/5	5/3	59.2 (37–82)	70.6 (58–86)	37.7 (25.5–42.7)	30.1 (22.7–44.4)	medial parapatellar	1, 3, 4
Alexander, 2015 [8]	RCT	USA	100	29/21	29/21	68.1 ± 8.5	68.1 ± 8.5	30.1 ± 4.6	30.1 ± 4.6	medial parapatellar	1, 2, 5, 6, 7,8
Aditya, 2015 [9]	retrospective study	USA	190	93/22	59/16	65 ± 9	61 ± 8	34 ± 6.4	34.3 ± 7.1	medial parapatellar	4
Chan, 2017 [15]	RCT	China	109	46/9	47/7	70.5 ± 8.2	70.4 ± 8.9	26.8 ± 1.2	26.5 ± 3.9	N	2, 7, 8
Rajesh, 2017 [16]	RCT	India	170	59/21	70/20	63.1 ± 8.8	60 ± 10.2	N	N	medial parapatellar	1, 2, 4, 5, 6
Li, 2019 [14]	RCT	China	76	30/8	30/8	43.76 (22–70)	43.76 (22–70)	23.78 ± 2.98	23.78 ± 2.98	medial parapatellar	1, 2
Carlo, 2019 [17]	RCT	Spain	85	61.4%/38.6%	68.3% /31.7%	73.8 ± 7.5	74.2 ± 8.2	30.6 ± 4.6	30.2 ± 5	N	2, 4, 5
Total			1546								

T, barbed suture group; C, traditional Suture group; N, not informed

1, capsule suture time (min); 2, Superficial infection; 3, Deep infection; 4, dehiscence; 5, Suture breakage; 6, Needle stick; 7, Hospital for Special Surgery score; 8, knee society score; *, The authors did not mention the data of TKA separately

**Table 2:** Risk of Bias Evaluation for the Retroactive Study (NOS)

Included Trials	Selection			Comparability		Outcome			total stars
	A	B	C	D	E	F	G	H	
Eickmann 2010 [13]	☆	☆	☆	☆	☆	☆	☆	☆	8
Jeremy 2012 [4]	☆	☆	☆	☆	☆	☆	☆	☆	8
Aditya 2015 [9]	☆	☆	☆	☆	☆☆	☆	☆		8

A, Representativeness of the exposed cohort; B, Selection of the non-exposed cohort; C, Ascertainment of exposure; D, Demonstration that outcome of interest was not present at start of study; E, Comparability of cohorts on the basis of the design or analysis; F, Assessment of outcome; G, was follow-up long enough for outcomes to occur; F, Adequacy of follow up of cohorts

(http://www.ohri.ca/programs/clinical_epidemiology/oxford.asp)

As to other complications, there were no significant differences (odds ratio (OR) 0.99 [95% confidence interval (CI), 0.41, 2.38]) (Test for overall effect, *p* = 0.99) in dehiscence between barbed and conventional sutures based on six studies. There was a significant difference (OR 0.16 [95% CI, 0.04, 0.72]) (Test for overall effect, *p* = 0.02) in needlestick injury based on three studies. In three studies, a significant difference was revealed in suture breakage between barbed and conventional sutures (OR 36.51 [95% CI, 7.06, 188.72]) (Test for overall effect, *p* < 0.0001) (Table 3).

**Table 3:** Meta-analysis Results for Other Outcome Measures

Outcome	Included Trials	Heterogeneity		Analysis Model	Meta-analysis Results	
		P	I^2^		P	OR (95%CI)
Dehiscence	6 [8, 10, 11, 13, 15, 17]	0.42	0%	OR, M-H, Fixed	0.99	0.99 (0.41,2.38)
Needle stick	3 [4, 8, 16]	0.79	0%	OR, M-H, Fixed	0.02	0.16 (0.04,0.72)
Suture breakage	3 [4, 16, 17]	0.90	0%	OR, M-H, Fixed	< 0.0001	36.51 (7.06,188.72)

## Discussion

Wound closure is an important aspect of TKA, because the joint capsule is highly stretched, and the integrity of the arthrotome closure must be maintained. Multiple techniques are available for the closure of the joint capsule after TKA. At present, either absorbable or non-absorbable suture is used to close the joint capsule. Absorbable synthetic braided suture is used to close the subcutaneous tissue and metal skin staple or suture is employed to suture the skin [22]. Conventional suture knots carry the potential risks of knot protrusion, subcutaneous palpable keloid, local microinfarction, breakage, sliding knots, *etc*. Barbed suture is a suture method that causes less tissue trauma and requires no knot-tying. Zaruby *et al* [23] augured that bidirectional barbed suture was safe and effective, and its strength and surrounding tissue reactions were comparable to those of absorbable suture. Chugaev *et al* [24] indicated that closure of joint capsule and subcutaneous adipose tissue with bidirectional knotless barbed sutures in primary TKA was safe and time-saving, because it did not cause occult blood loss or the occurrence of infection. Shermak *et al* [25] found barbed sutures were associated with greater difficulty in the healing of the knee extensor mechanism and higher complication rates when fascia and subcutaneous tissues were sutured. The reason is that barbed sutures increase suture surface area compared to the conventional sutures, thereby enlarging suture tunnels and increasing the incidence of incision complications [26]. Similar incidences of superficial and deep infections were observed between barbed sutures and conventional sutures in TKA. However, the risk of using barbs was associated with a more likelihood of suture breakage.

In 2019, Thacher* et al* [27] found that superficial skin closure with barbed sutures resulted in an increased incidence of wound dehiscence in total hip arthroplasty. However, barbed continuous sutures were associated with shorter time for wound closure than the standard sutures, though barbed sutures showed less benefit in suture switching and suture failure [8]. Fewer suture switches with barbed suture not only reduced overall operative time, but also more effectively avoided needlestick injuries. Although the barbed suture instruments were more expensive than the conventional suture instruments, the latter is a time-consuming process [12]. Gililland *et al* [4] concluded that the average estimated time for wound closure with barbed suture was shorter. Therefore, the cost of two suture techniques are generally the same. Smith *et al* [10] concluded that barbed sutures saved $ 55 on average in each arthroplasty.

Watertightness of the knee joint is a different aspect of repair integrity from arthrotome closure. In a cadaveric study, Kobayashi *et al* [28] suggested that the use of barbed sutures appeared appropriate for maintaining maximum watertightness after knee capsule closure, and could improve resistance to early mobilization protocols, and achieve early deep knee flexion. Our results showed similar HSS and KSS scores with the use of two suture techniques. We believe that barbed suture may exert an effect on early deep knee flexion, but no effect on range of motion of the knee 3 to 12 months after operation.

Our study has several limitations. First, there was a bias due to selective inclusion and reporting of outcomes and analyses in systematic reviews of RCTs. Second, poor-quality and poorly reported RCTs might well yield biased results. Third, in some trials, no clearly defined adverse events were reviewed, nor specified complications were selected for inclusion. Fourth, different rating scales and definitions among non-standardized rating scales used for rating entity or outcomes were given by different rating agencies in non-RCTs. Fifth, the definitions of high-quality studies might vary considerably, which might be related to the perception of investigators.

## Conclusions

In TKA, both barbed and conventional sutures have the similar incidences of superficial and deep infection, Hospital for Special Surgery Knee Score, Knee Society Score, and wound dehiscence. Barbed suture has higher incidence of suture breakage, shorter suture time, and less needle stick injury. However, the total cost of wound closure may vary substantially, because the time and cost for anesthesia, time for wound closure procedures, and cost of suture instruments are different, depending on countries, regions, hospital, and even surgeon’s skills, among others.

## Data Availability

All data generated or analyzed during this study are included in this published article.

## Abbreviations

TKA: Total Knee Arthroplasty
RCT: Randomized controlled trial
NOS: Newcastle-Ottawa Scale
HSS: Hospital for Special Surgery Knee Score
KSS: Knee Society Score
ROM: Range of Motion
OR: Odd ratio
SD: Standard deviation
CI: Confidence interval
